# Cardiorespiratory Fitness in Spanish Youth: The Roles of Sex, Age, Body Composition, and Healthy Lifestyle Habits in Cor-School Study

**DOI:** 10.3390/children12050581

**Published:** 2025-04-29

**Authors:** Saül Aixa-Requena, Álvaro Pano-Rodríguez, Vicenç Hernández-González, Enric Conesa-Milian, Abraham Batalla-Gavaldà, Jose Vicente Beltrán-Garrido, Isaac López-Laval, Francisco Corbi, Rosa Arnau-Salvador, Joaquín Reverter-Masia

**Affiliations:** 1 Human Movement Research Group (RGHM), University of Lleida, Plaça de Víctor Siurana, 25003 Lleida, Spain; alvaro.depano@udl.cat (Á.P.-R.); vicenc.hernandez@udl.cat (V.H.-G.); enric.conesa@udl.cat (E.C.-M.); presidencia@cofcastellon.org (R.A.-S.); joaquim.reverter@udl.cat (J.R.-M.); 2 Faculty of Education, Psychology and Social Work, Department of Specific Didactics, University of Lleida, 25003 Lleida, Spain; 3 University School of Health and Sport (EUSES), Universitat Rovira i Virgili, 43870 Amposta, Spain; a.batalla@euseste.es; 4 Department of Education and Specific Didactics, Faculty of Humanities and Social Sciences, Universitat Jaume I, Castellón de la Plana, 12006 Castellón, Spain; 5 Physical Exercise and Performance Research Group, Universidad Cardenal Herrera-CEU, CEU Universities, Castellon de la Plana, 12006 Castellón, Spain; jose.beltrangarrido@uchceu.es; 6 Department of Education Sciences, School of Humanities and Communication Sciences, Universidad Cardenal Herrera-CEU, CEU Universities, Castellon de la Plana, 12006 Castellón, Spain; 7 Faculty of Health and Sport Science, Department of Physiatry and Nursing, University of Zaragoza, 50009 Huesca, Spain; isaac@unizar.es; 8 National Institute of Sport and Physical Education (INEFC), University of Lleida, 25003 Lleida, Spain; fcorbi@gencat.cat; 9 Official College of Pharmacists of Castellón, 12004 Castellón, Spain

**Keywords:** physical activity, youth health, cardiorespiratory fitness, body composition, gender differences

## Abstract

**Background/Objectives**: Cardiorespiratory fitness (CRF) is a key indicator of current and future health in children and adolescents, and is influenced by both physiological and behavioral factors. This study examined the associations between CRF, body composition, and health-related habits in 685 children and adolescents (aged 8–16 years) who regularly participated in extracurricular sports programs in northeastern Spain. **Methods:** Participants underwent anthropometric assessments, completed a 20 m shuttle run test to evaluate CRF, and filled out validated questionnaires on physical activity levels, sleep quality, and adherence to the Mediterranean diet. **Results:** Significant sex differences were observed, with boys achieving higher CRF performance (*p* < 0.001), primarily associated with greater muscle mass (*p* < 0.001) and lower body fat percentage (*p* < 0.001). Age positively correlated with improved CRF (*p* < 0.001), whereas higher body mass index (*p* = 0.003) and fat mass (*p* = 0.020) negatively impacted fitness outcomes. **Conclusions:** These findings underscore the critical role of physical activity and optimal body composition in promoting cardiorespiratory fitness among youth, emphasizing the importance of targeted interventions to foster healthy behaviors from early childhood. Future research should adopt longitudinal designs to better elucidate the dynamic interactions between lifestyle factors, body composition, and physical fitness throughout childhood and adolescence.

## 1. Introduction

It is well established that regular physical activity provides numerous health benefits, particularly when initiated at an early age, due to its immediate and long-term impact [1,2,3,4]. Currently, physical inactivity is a major societal issue, becoming increasingly concerning in pediatric populations [4,5]. Childhood and adolescence represent critical developmental stages during which individuals are highly receptive to external stimuli and lifelong habits begin to form [6].

A recent report by the Non-Communicable Diseases Risk Factor Collaboration (NCD-RisC) (2024) indicated that in 2022, 18.6% of children and adolescents aged 5–19 years were overweight, including 7.6% classified as obese, marking a significant increase compared to previous decades [7]. In 2019 in Spain, the ALADINO study reported that among children aged 6 to 9 years, 23.3% were overweight and 17.3% were obese, totaling 40.6% of children with excess weight [8]. These figures underscore the growing global and national concern regarding childhood overweight and obesity, commonly assessed through body mass index (BMI), which can severely affect children’s health by increasing the risk of hypertension, type 2 diabetes, elevated triglycerides, bone fractures, joint problems, heart disease, poor mental health, sleep disturbances, and decreased overall life satisfaction [9,10,11,12]. A contributing factor to these adverse health outcomes is the concurrent rise in physical inactivity among children, which exacerbates risks associated with obesity and poor health [13].

Rates of physical inactivity among children are increasing at an alarming pace, and this issue becomes even worse during adolescence. Recent reports indicate that this trend is declining over time, with 81% of adolescents not reaching the minimum recommended daily amount of physical activity [4], a situation expected to further decline in the coming years [14]. Adolescence is already a complex developmental stage, characterized by significant social, contextual, and hormonal changes, contributing to reduced physical activity during leisure time [15].

Both physical activity and cardiorespiratory fitness (CRF), defined as the ability of the circulatory and respiratory systems to supply oxygen to skeletal muscle during prolonged moderate-to-vigorous exercise [16], are predictors of potential diseases and mediators of other health parameters, such as sleep quality [17]. Closely linked to these factors, BMI influences CRF outcomes, with higher values often indicating reduced fitness and elevated risk of future health issues, underscoring their interconnected roles in youth well-being [18,19]. Furthermore, research indicates that CRF generally improves during childhood and adolescence, influenced by factors such as growth, maturation, and increased physical activity [18]. As children develop, increases in fat-free mass significantly contribute to improvements in CRF [19]. Additionally, participation in organized sports has been associated with higher physical activity levels, positively influencing CRF during these developmental stages [20].

Regular physical activity and good CRF have also been shown to facilitate better sleep patterns, addressing a common issue among adolescents [21,22]. Indeed, factors such as diet and overall quality of life significantly influence daily physical activity, sleep quality, and CRF. For instance, unhealthy dietary patterns and low physical activity levels have been associated with an increased risk of overweight and obesity in adolescents [23]. Additionally, studies have shown that poor eating habits negatively impact sleep quality in children and adolescents [24]. Conversely, adherence to healthy dietary patterns, such as the Mediterranean diet, has been positively associated with improved health-related quality of life, mediated by physical fitness in adolescents [25]. These findings underscore the interconnectedness of diet, quality of life, and various aspects of health in young populations.

This article presents findings from a cross-sectional analysis conducted in 2024, under the framework of the COR-School project [26], focusing primarily on CRF as the dependent variable, and exploring its associations with independent factors such as age, maturation stage, sex, physical activity, dietary habits, sleep quality, and body composition among Spanish school-aged children regularly engaged in sports activities. Unlike prior studies that have examined these variables in isolation or via secondary datasets, our work uniquely combines primary, first-hand measurements of fitness, body composition, and multiple lifestyle behaviors in a single, large cohort of 685 Spanish youth, providing novel, population-specific benchmarks. Although CRF is the central outcome, the interplay among these factors may be complex and bidirectional, given that higher CRF, regular physical activity, and healthier dietary patterns can also positively influence other health-related parameters, such as sleep quality and body composition. Considering these relationships, we hypothesized that higher levels of physical activity, better adherence to the Mediterranean diet, and adequate sleep would be associated with greater cardiorespiratory fitness and healthier body composition.

## 2. Materials and Methods

### 2.1. Participants

A total of 685 children and adolescents (mean age 11.67 ± 1.65 years), aged between 8 and 16 years, recruited from extracurricular sports programs located in provinces in northeastern Spain, participated in this study. An invitation letter was sent to all schools and sports clubs in the region, and all institutions that responded positively were included. The sample consisted of 265 girls and 420 boys, all of whom completed the study. As part of the COR-School project [26], a minimally active cohort was targeted to ensure stability of field-test performance and to mitigate risks associated with high-intensity activities [27,28].

The participants were informed about this study’s objectives, as well as the potential risks and benefits associated with their participation in this study. Informed consent was obtained from all participants and their legal guardians prior to participation, along with a detailed family medical history form to evaluate health risks and suitability for testing. Individuals were informed that they could withdraw from this study at any time without providing a reason, and that their data would be permanently deleted upon withdrawal. Given that this study included a maximal exercise test as a key variable, participants with any known cardiac or health issues that might prevent them from sustaining maximal effort were excluded.

### 2.2. Procedures

Recruitment was carried out by contacting schools and sports organizations across northeastern Spain, including both rural areas and major cities from South Catalonia and North Valencia. After providing comprehensive information about the project and obtaining signed informed consent and family medical history forms, testing dates were scheduled.

Participants were not required to follow dietary restrictions before testing. They were encouraged to maintain their regular diet but advised to avoid heavy meals immediately before the assessments. Light eating and hydration were permitted throughout the testing day, except during the 20 m shuttle run test (20 m SRT). The only restriction imposed was on moderate to vigorous physical activity (MVPA) during the 48 h prior to testing.

The variables collected included age, anthropometric parameters, cardiovascular fitness, maturation status, and health-related habits among youth. Maturation status and health-related habits were assessed using validated questionnaires, which participants completed electronically via tablets. Responses were automatically stored in a secure database for subsequent processing and analysis [26]. Later, participants received an individualized summary report of their results after data processing and analysis had concluded.

All procedures were approved on 15 December 2020, by the Catalan Sports Research Ethics Committee (30/CEICGC/2020), and complied with the latest revised version of the Declaration of Helsinki [29].

### 2.3. Variables and Instruments

#### 2.3.1. Age and Maturation

Age was recorded on the day of testing in years with one decimal place. Biological maturation was assessed using a brief self-reported questionnaire with illustrations and explanations [30] of Tanner’s stages of maturation [31], allowing participants to select the stage closest to their current status.

#### 2.3.2. Anthropometric Variables

Anthropometric measurements were conducted following the standards of the International Society for the Advancement of Kinanthropometry (ISAK) [32]. Body mass was measured using a digital scale (Seca 877, Hamburg, Germany; accuracy ±0.1kg), and height was assessed with a portable stadiometer (Seca 213, Hamburg, Germany; accuracy ± 0.1cm). BMI, widely used as a predictor of health-related issues in children [33] and adolescents [34], was calculated by dividing body mass in kilograms by the square of height in meters (kg/m^2^). Skinfold thickness was measured with a calibrated skinfold caliper (Baty International, Harpenden, UK; accuracy ± 0.2mm) at standard anatomical sites, and a non-elastic anthropometric tape (Lufkin W606PM, Apex Tool Group, Sparks Glencoe, MD, USA) was used for required girths. Estimates of fat and muscle mass percentages were calculated using ISAK-approved equations appropriate for the study population [32].

#### 2.3.3. Cardiorespiratory Fitness

CRF was assessed using the 20 m SRT, also known as the Léger test or course navette [35]. This test is part of the ALPHA-Fitness battery developed for the HELENA project, specifically designed to evaluate physical fitness in children and adolescents [36,37]. It has been previously validated as a reliable measure of CRF for these age groups [38], and a recent international consensus identified it as the most widely used and recommended field-based test for assessing aerobic capacity in youth across Europe, due to its feasibility, standardization, and scalability [39].

The participants were required to run between two parallel lines placed 20 m apart, maintaining a pace dictated by an audio signal that increased progressively each minute. The test commenced at a speed of 8 km/h and increased by 0.5 km/h per minute. The test concluded when participants could no longer maintain the required pace (i.e., when they failed to reach the line in synchronization with the audio signal). The final stage successfully completed by each participant was recorded as the test result. Standardization was ensured by adhering to the established protocols for the ALPHA-Fitness battery [36].

#### 2.3.4. Daily Physical Activity

Daily physical activity was assessed using the Spanish version of the Physical Activity Questionnaire (PAQ), previously tested and validated for children (PAQ-C) [40] and adolescents (PAQ-A) [41]. The PAQ questionnaires have demonstrated acceptable reliability and validity in youth populations when administered under appropriate conditions [40,41,42,43,44].

#### 2.3.5. Sleep Quality

Sleep quality was assessed using the Pittsburgh Sleep Quality Index (PSQI) [45]. The PSQI comprises seven Likert-type items, providing a total score ranging from 0 to 21 points, with higher scores indicating poorer sleep quality. The Spanish version of the PSQI has been validated and demonstrated good reliability in pediatric populations [46].

#### 2.3.6. Adherence to the Mediterranean Diet

Adherence to the Mediterranean diet, which is associated with better overall and cardiovascular health [47], was assessed using the KIDMED questionnaire [48]. The KIDMED questionnaire was specifically designed for pediatric populations and has demonstrated validity and reliability in its Spanish language version [49]. This questionnaire is a Food Frequency Questionnaire (FFQ) that assesses the frequency of consumption of various food items associated with the Mediterranean diet [48].

To enhance the accuracy of responses, all questionnaires were administered in a supervised setting. Two graduates in physical activity and sports sciences were present during the administration to assist participants, clarify any doubts, and ensure comprehension, thereby minimizing potential biases associated with self-reporting.

### 2.4. Statistical Analysis

To assess normality, the Kolmogorov–Smirnov test was used due to the large sample size (N = 685), although visual inspections of Q-Q plots and histograms of residuals were also performed. Group comparisons were performed using Student’s *t*-test for normally distributed variables and the Mann–Whitney U test for non-normally distributed variables. Categorical variables were analyzed using the chi-square test, with post hoc analyses based on adjusted residuals to examine differences between sexes.

A linear regression model was used to estimate the CRF level score, stratified by sex and adjusted for age, BMI, fat mass percentage, and muscle mass. The meters run in the 20 m SRT test was included as the dependent variable. Since the only categorical predictor (sex) had two levels, no multiple comparison adjustments were applied.

Model fit was assessed using the log-likelihood ratio test. Statistical significance was set at α < 0.05. Data are reported as mean (SD), median (Mdn) with interquartile range (IQR: Q1–Q3), or frequency, depending on variable type.

All analyses were performed using JAMOVI for Mac [50] (version 2.6.44) and the GAMLj module for generalized linear modeling [51].

## 3. Results

The descriptive characteristics of the study population are shown in Table 1.

Statistically significant differences between sexes were reported for height (*p* = 0.012), waist (*p* < 0.001), fat mass percentage (*p* < 0.001), muscle mass (*p* < 0.001), pubertal status (*p* = 0.043), and physical activity levels (*p* < 0.001).

However, post hoc tests using adjusted residuals for pubertal status revealed no significant differences between sexes, as the adjusted residuals for all categories (I, II, III, IV, and V) remained below the significance threshold (|z| < 1.96). These findings suggest that men and women were of similar pubertal status.

The parameter estimates (coefficients) of the general linear model are shown in Table 2.

A statistically significant effect of the sex variable on the meters run by the participants in the 20 m SRT test was observed. Boys ran more meters than girls (b = 263.09 95% CI [182.32, 340.27], t = 6.33, *p* < 0.001).

A statistically significant effect of the age variable on the meters run by the participants in the 20 m SRT test was observed. The older the participants, the greater the number of meters run by the participants in the physical fitness test (b = 112.32 95% CI [65.05, 162.33], t = 4.44, *p* < 0.001).

A statistically significant effect of the BMI variable on the meters run by the participants in the 20 m SRT test was observed. Higher BMI levels were associated with fewer meters run by participants in the physical fitness test (b = −136.27 95% CI [−220.38, −41.52], t = −3.00, *p* = 0.003).

A statistically significant effect of the fat mass variable on the meters run by the participants in the 20 m SRT test was observed. Higher levels of fat mass were associated with fewer meters run by participants in the physical fitness test (b = −90.15 95% CI [−169.21, −16.98], t = −2.33, *p* = 0.020).

A statistically significant effect of the muscle mass variable on the meters run by the participants in the 20 m SRT test was observed. Higher levels of muscle mass were associated with more meters traveled by participants in the physical fitness test (b = 79.83 95% CI [5.25, 156.03], t = 2.08, *p* = 0.038).

## 4. Discussion

The findings of this study provide valuable insights into the relationship between cardiorespiratory fitness, body composition, and health-related habits among school-aged children and adolescents. The results indicate significant differences between boys and girls in key anthropometric measures, physical activity levels, and fitness-related parameters, highlighting the complex interplay among biological, maturational, and lifestyle factors in determining physical fitness outcomes.

### 4.1. Sex Differences

Consistent with previous research, boys outperformed girls in the 20 m SRT, achieving significantly greater distances [52]. This advantage is likely driven by boys’ higher muscle mass [53], which enhances aerobic capacity and endurance [54], and their lower fat percentage, given that greater adiposity impairs shuttle-run performance [55]. Interestingly, despite these sex-related differences in physical fitness and body composition, no significant differences were observed in pubertal status between boys and girls, as indicated by post hoc analyses using adjusted residuals. This suggests that, at this specific development stage, disparities in performance and body composition may be more attributable to inherent physiological sex differences (such as muscle and fat distribution) rather than differences in biological maturation [56]. However, it is important to note that biological maturation may play a more prominent role in explaining physiological differences at other stages of development [57].

### 4.2. Impact of Age

As shown in the results, age is a significant predictor of physical fitness performance [58], with older participants covering significantly more distance in the 20 m SRT [52]. This finding aligns with the expected improvements in aerobic capacity and musculoskeletal strength associated with increasing age and maturation [59]. The progressive increase in muscle mass with age likely contributes to enhanced exercise performance, as evidenced by the positive association between muscle mass and performance in the 20 m SRT [54].

### 4.3. Impact of Body Composition

BMI and fat mass were both negatively associated with physical fitness performance. Higher BMI was linked to significantly reduced distance covered in the 20 m SRT, suggesting that greater body weight, particularly when attributable to increased fat mass, may negatively affect CRF [60,61]. As a predictor of health risks in children, elevated BMI also signals potential long-term consequences, such as cardiovascular disease and diabetes, highlighting its dual role in fitness and overall health [14]. Similarly, higher fat mass was associated with poorer performance, reinforcing the adverse impact of excess adiposity on physical endurance and aerobic capacity [62]. Conversely, muscle mass demonstrated a positive relationship with performance, underscoring the importance of muscular strength and lean body mass in supporting physical fitness among youth [63]. Those results are in line with other studies in Spain [64] and other countries like the United States [65], China [66], and Brazil [67], where a higher BMI was related to poor cardiorespiratory fitness.

Given these findings, it is essential to consider targeted educational strategies and school-based intervention programs aimed at promoting physical activity and improving body composition, particularly among children and adolescents with higher BMI or fat mass. Schools represent a key setting for the implementation of structured and inclusive physical education programs that not only encourage regular movement and fitness development, but also foster positive attitudes toward physical activity [68]. Early engagement in such programs may be crucial to prevent the consolidation of sedentary behaviors and to reduce long-term health risks, supporting both physical fitness and overall well-being throughout development [69].

### 4.4. Physical Activity and Lifestyle Factors

Significant differences were observed between boys and girls in physical activity levels, as measured by the Physical Activity Questionnaire (PAQ) [70]. Higher activity levels among boys may partially explain their superior performance in the 20 m SRT [71] and their more favorable body composition profile [72]. These findings suggest that sex-related differences in cardiorespiratory fitness and body composition may not be exclusively attributable to physiological characteristics such as fat and muscle distribution, but also to behavioral factors like physical activity engagement [73]. Higher levels of physical activity are known to promote lean mass, reduce fat accumulation, and enhance aerobic capacity, potentially mediating the observed body composition and performance differences [74]. This highlights the importance of considering both biological and behavioral dimensions when interpreting sex differences in physical fitness during childhood and adolescence.

In contrast, no significant differences were found between boys and girls regarding adherence to the Mediterranean diet (KIDMED) or sleep quality (PSQI), suggesting that other behavioral and environmental factors may have a greater influence in shaping sex-based differences [75,76]. However, given BMI’s role as a health predictor, the potential influence of diet and sleep on broader outcomes, such as metabolic health or obesity risk, merits further exploration in this population [24,25].

### 4.5. Strengths, Limitations, and Future Research

While previous studies and meta-analyses have addressed the associations between individual lifestyle factors (such as physical activity [5,77], diet [23,78], and sleep [21,79]) and indicators of physical fitness or health outcomes, these elements have often been examined separately or based on secondary data. The present study contributes to the existing literature by providing original research, with a large and diverse sample size (N = 685), that simultaneously assesses multiple lifestyle variables and their relationship with cardiorespiratory fitness and body composition using primary data collected directly from a large cohort of children and adolescents. This comprehensive and integrative approach allows for a more complete and ecologically valid understanding of how behavioral and physiological dimensions interact during youth, offering valuable insights for both research and intervention design.

Nevertheless, several limitations should be acknowledged. First, the cross-sectional design precludes establishing causal relationships among variables. Second, the reliance on self-reported measures of physical activity, dietary habits, and sleep quality may introduce recall bias, social desirability bias, and misclassification. Although test–retest reliability for such instruments in youth often falls within acceptable ranges (ICC ≈ 0.60–0.80), evidence of their criterion validity is limited or mixed, and some domains exhibit poor reliability [80]. Additionally, unmeasured factors such as socioeconomic status and access to sports facilities might have influenced observed differences in physical fitness and body composition.

Future research should examine longitudinal trends in physical fitness and body composition and investigate the role of environmental and socioeconomic factors in shaping health outcomes among youth.

## 5. Conclusions

In conclusion, this study highlights the significant influence of sex, age, body composition, and physical activity levels on physical fitness in school-aged children and adolescents. Boys demonstrated superior performance in the 20 m SRT, closely associated with higher muscle mass and lower fat mass. Age-related improvements in aerobic fitness further emphasize the contribution of maturation and musculoskeletal development. These findings underline the importance of promoting regular physical activity and healthy body composition as essential strategies to enhance CRF and overall health among youth.

## Figures and Tables

**Table 1:** Descriptive characteristics of the study population.

Variable	All (n = 685)	Girls (n = 265)	Boys (n = 420)	*p* Values
Age (years) ^a^	11.67 ± 1.65	11.61 ± 1.68	11.70 ± 1.63	0.471
Height (m) ^a^	1.53 ± 0.12	1.52 ± 0.11	1.54 ± 0.13	**0.012**
Weight (kg) ^a^	46.81 ± 12.01	46.37 ± 11.16	47.09 ± 12.52	0.445
BMI (kg·m^−2^) ^a^	19.69 ± 3.14	19.90 ± 3.09	19.57 ± 3.17	0.433
Waist (cm) ^b^	65.65 ± 10.00	64.00 ± 9.50	66.40 ± 10.65	**<0.001**
Fat mass (%) ^a^	22.89 ± 5.96	25.36 ± 4.81	21.05 ± 6.07	**<0.001**
Muscle Mass (kg) ^a^	34.09 ± 8.14	32.45 ± 6.83	35.31 ± 8.81	**<0.001**
Tanner stage, I–V ^c^	105/205/170/119/20	33/68/75/53/10	72/137/95/66/10	**0.043**
PAQ-C/A (MET-min·week^−1^) ^a^	2.99 ± 0.60	2.84 ± 0.56	3.09 ± 0.60	**<0.001**
KIDMED (AU) ^a^	6.24 ± 2.45	6.30 ± 2.56	6.19 ± 2.38	0.575
PSQI, 0–21 (AU) ^b^	3.00 ± 2.00	3.00 ± 2.00	3.00 ± 2.00	0.247

Values in bold indicate statistically significant results (*p* < 0.05). Abbreviations: BMI: Body mass index. Waist: Waist circumference. PAQ-C/A: Physical activity questionnaire-children/adolescent. KIDMED: Adherence to the Mediterranean questionnaire. PSQI: Pittsburgh Sleep Quality Index. ^a^ Data are presented as mean (SD), and differences between boys and girls were examined by analysis of variance. ^b^ Data are presented as median (IQR), and differences between boys and girls were examined by independent-sample Mann–Whitney U tests. ^c^ Data are presented as frequency (%), and differences between boys and girls were examined by independent-sample chi-square test.

**Table 2:** Parameter estimates (coefficients) of the general linear model.

Effect	Estimate	SE	Lower 95% CI	Upper 95% CI	t	*p*
(Intercept)	1270.85	17.85	1236.68	1304.49	71.22	**<0.001**
Sex (B–G)	263.09	41.57	182.32	340.27	6.33	**<0.001**
Age (years)	112.32	25.32	65.05	162.33	4.44	**<0.001**
BMI (kg·m^−2^)	−136.27	45.46	−220.38	−41.52	−3.00	**0.003**
Fat mass (%)	−90.15	38.65	−169.21	−16.98	−2.33	**0.020**
Muscle mass (kg)	79.83	38.33	5.25	156.03	2.08	**0.038**

Values in bold indicate statistically significant results (*p* < 0.05). Abbreviations: B: Boys, G: Girls. BMI: Body mass index.

## Data Availability

The datasets generated during and/or analyzed during the current study are available from the corresponding author on reasonable request.
